# Study design and methods of the Ansan Geriatric Study (AGE study)

**DOI:** 10.1186/1471-2377-9-10

**Published:** 2009-02-24

**Authors:** Changsu Han, Sangmee Ahn Jo, Nan Hee Kim, Inho Jo, Moon Ho Park

**Affiliations:** 1 Department of Psychiatry, Korea University Medical College, 516, Gojan-dong, Danwon-gu, Ansan-shi, Gyeonggi-do 425-707, Republic of Korea; 2 Center for Biomedical Sciences, Biomedical Brain Research Center, National Institute of Health, 194, Tongil-lo, Eunpyung-gu, Seoul 122-701, Republic of Korea; 3 Division of Endocrinology and Metabolism, Department of Internal Medicine, Korea University Medical College, 516, Gojan-dong, Danwon-gu, Ansan-shi, Gyeonggi-do 425-707, Republic of Korea; 4 Department of Molecular Medicine, Ewha Womans University Medical School, 911-1, Mok-dong, Yangchun-gu, Seoul 158-710, Republic of Korea; 5 Department of Neurology, Korea University Medical College, 516, Gojan-dong, Danwon-gu, Ansan-shi, Gyeonggi-do 425-707, Republic of Korea

## Abstract

**Background:**

The overall objective of the Ansan Geriatric Study (AGE study) was to describe the prevalence, incidence, and related risk factors for geriatric diseases in elderly Koreans.

**Methods/Design:**

The AGE study was designed as a population-based prospective cohort study on health, aging, and common geriatric diseases of elderly Koreans aged 60 to 84 years. The inception cohort was recruited in May 2002. The first-wave and second-wave studies were performed using uniform and structured procedures. At the screening study, 2,767 participants were enrolled. Participants (1391 in the first wave study and 841 in the second wave study) were recruited and completed the evaluation. The prevalence of geriatric disease and related factors in elderly Koreans were estimated.

**Discussion:**

Here, we report the design and sampling participants, measurement tools, and characteristics of the AGE study. This cohort study will allow a detailed study of the longitudinal comprehensive data on health information of elderly Koreans, thereby contributing to policy formulation and planning of health, welfare management, and other social services in Korea.

## Background

More than one-quarter of the world's population will be over the age of 60 by the year 2010 [1]. As in most other countries, the proportion of elderly people in Korea is increasing every year due to decreased birth rates and increased longevity. The proportion of those 65 years and older in Korea was approximately 8.3% in 2003 and is expected to rise to 15% in 2019 [2]. As Korea will have the highest proportion of elderly people in the world by 2050, public health concerns about geriatric diseases in Korea have taken on greater importance. There are increasing demands for national policies and programs to deal with problems affecting the elderly. Thus, there is a need for research focusing on age-related conditions that contribute significantly to chronic illness and disability in elderly Koreans.

Although there have been several Western elderly cohort studies such as the Baltimore Longitudinal Study on Aging in the USA [3], the Framingham study in the USA [4], the Rotterdam elderly study in the Netherlands [5], and the Canadian Study on Health and Aging in Canada [6], there have been a few longitudinal cohort studies with elderly Asians. The Honolulu Asia Aging Study [7] investigated men only, whereas longitudinal cohort studies on elderly Koreans including the Hallym Aging Study (HAS) [8], the Gwangju dementia and mild cognitive impairment study (GDEMCIS) [9], and the Korean Longitudinal Study on Health and Aging (KLoSHA) [10] had some methodological problems; a precise neuropsychological test was not performed, the subjects were recruited without population-based random sampling, or there was only cross-sectional evaluation.

The Ansan Geriatric study (AGE study) was designed to establish a prospective population-based cohort of subjects to study prevalence, incidence, and related risk factors for geriatric diseases and to obtain comprehensive information on public health and functional status in elderly Koreans. Here, we describe the design, sampling, participants, baseline measures, and sample characteristics of this study.

## Methods

### Study design and sampling population

As part of a baseline investigation to construct an elderly cohort in Korea, we sampled the elderly population living in Ansan-city, located 25 miles (40 km) southwest of Seoul and now considered a part of the metropolitan area of Seoul. According to the 2002 census, Ansan is an urban community with an elderly population of 36,735, aged 60 to 84 years. We assumed that the prevalence of major geriatric disease such as dementia would be 10–13% in the elderly Korean [11,12]. We wish to determine this prevalence to within about 1%, that is have a confidence interval of width 2% with 95% confidence, the number of subjects required is approximately 3000 [13].

The sampling protocol and design of the AGE study for a baseline investigation (screening study) have been well described previously [14-16]. Briefly, to enroll participants, we used a list of telephone numbers obtained from local telephone companies because the community has a high percentage of telephone subscribership. We conducted a two-stage cluster sampling based on information from the governing district, called "dong," and from the telephone directory, obtaining information on age and sex distribution from the 2002 census. To acquire a probability sample proportional to the age- and gender-specific population structure of the target population, a random sampling comprised 15,392 persons (41.9%) aged 60 to 84 years was selected. A letter inviting participation in this study was initially sent to all 15,392 individuals and they were additionally contacted at least three times either by telephone or were visited at home to confirm their eligibility. After the exclusion of individuals who were determined ineligible for interview, a total of 4,404 eligible individuals (12% of the target population or 28.6% of the stratified random sample) underwent baseline evaluation. After excluding persons who provided incomplete data or were ineligible for a probability sample proportional to the age- and gender-specific structure of the target population, the final sample comprised 2,767 elderly Koreans representative of the target population.

From the baseline investigation database, we constructed a first-wave study with a probability sample proportional to the age- and gender-specific population as described previously [17,18]. A total of 1,391 subjects (595 men and 796 women) were randomly recruited between September 2004 and March 2006. Recruits participated in a comprehensive health examination and on-site interviews at the Geriatric Health Clinic Research Institute, Korea University Ansan Hospital. The first-wave study participants were evaluated with clinical and neuropsychological examinations and were subjected to genotyping for the APOE ε4 polymorphism. In this elderly population, some participants did not complete follow-up at all intervals because of refusal, relocation, or death.

The follow-up assessment occurred from April 2006 to January 2008 (second-wave study; 25.61 ± 5.08 months), with uniform, structured follow-up evaluations by examiners that were blinded to the previously collected data. A total of 841 subjects were randomly recruited from the first-wave study. Variations in follow-up time, which ranged from 11 to 45 months, resulted from variations in response time to one or several invitations. The second-wave data collection was essentially the same as the first-wave study. Future follow-up assessments will be performed at regular intervals. The participants of the AGE study will be re-examined using the same procedures used in the previous assessments.

Procedures were in accordance with institutional guidelines and were approved by an institutional review committee. Informed written consent for participation was obtained from each individual and the study protocol was approved by the institutional review board of the AGE study.

### Neuropsychological examination

During the first-wave study, a comprehensive and validated battery of neuropsychological tests was performed by trained neuropsychologists. The Word Fluency Test, the 15-item Korean version of the Boston Naming Test, the Mini-Mental State Examination in the Korean version (MMSE-KC), the Word List Memory test, the Constructional Praxis test, the Word Delayed Recall test, the Word Recognition test, the Construction Recall test, and the Korean version of the Consortium to Establish a Registry for Alzheimer's Disease (CERAD) neuropsychological assessment battery of tests (CERAD-K) [19] were administered. Additionally, we also administered part of the series of neuropsychological tests in the Seoul Neuropsychological Screening Battery (SNSB) [20] to assess other specific cognitive domains: The Digit Span Test and Calculation for attention, and the Contrasting Program, Go-No-Go Test, Fist-Edge-Palm, Alternating Hand Movement Test, and Alternating Square and Triangle Test for frontal executive functions. The total score for the CERAD neuropsychological battery was used as an index of the overall level of cognitive functioning of the participants [21]. The Clinical Dementia Rating Scale (CDR) was also administered [22]. The CDR was based on a structured caregiver interview. Scores were obtained in six different functional domains (memory, orientation, judgment and problem solving, community affairs, home and hobbies, and personal care). A variety of scores can be calculated but for the purposes of this study we used the "sum of boxes" score which was the arithmetic sum of the six subscores [23]. Neither instrument was used in any algorithmic way to determine clinical diagnosis and instrument scores could diverge from clinical diagnosis.

Dementia was defined according to the diagnostic features of dementia given in the Diagnostic and Statistical Manual of Mental Disorders, fourth edition (DSM-IV) [24]. The sub-diagnosis of possible or probable Alzheimer' disease (AD) was based on the National Institute of Neurological and Communicative Disease and Stroke-Alzheimer's Disease and Related Disorder Association criteria (NINCDS-ADRDA) [25]. Vascular dementia (VD) was diagnosed according to the National Institute of Neurological Disorders and Stroke-Association Internationale pour la Recherche et l'Enseignement en Neurosciences (NINDS-AIREN) criteria possible VD [26]. The Hachinski ischemic score was determined to estimate the vascular contribution to cognitive decline [27].

Mild Cognitive Impairment (MCI) was diagnosed on the basis of the International Working Group on Mild Cognitive Impairment [28] as follows. Objective cognitive impairment was assessed using tasks in four specific cognitive domains: Memory, language function, visuospatial function, and frontal executive function. Briefly, memory was assessed with the Word List Recall [19] and Constructional Recall tests [19]. Language function was assessed with the modified Korean version of the Boston Naming Test [29]. Visuospatial function was assessed with the Constructional Praxis test where four line drawings of figures with increasing complexity are presented to the subjects for copying [30]. Frontal executive function was assessed with the motor and fluency domains. The motor domain was assessed by the Contrasting Program test, the Go-No-Go test, the Fist-Edge-Palm test, the Alternating Hand Movement test, the Alternating Square and Triangle test, and the Luria loop test [31]. Motor impairment was defined as the condition where three or more tests showed abnormal results. The fluency domain was assessed by category fluency of the Korean version of the Controlled Oral Word Association Test [32]. Cognitive impairment was defined as an average composite score 1.0 SD below the age-, gender-, and education-specific mean of non-demented persons on the respective tests to conform to published criteria [33,34]. Furthermore, for the need for supervision or external help in performing any basic and instrumental activities of daily living (functional dependency due to physical impairment was not considered) was evaluated for all participants. Activities of daily living were assessed by the Barthel ADL [35] and the Korean Instrumental ADL [36], and the absence of DSM-IV clinical criteria for dementia was also assessed [24]. Although the consensus criteria also include confirmation of subjective memory complaints, this criterion was not included in the AGE study because its use in elderly Koreans [37] and population-based investigations has been questioned [38,39]. The clinical diagnosis of MCI was classified according to three subtypes: Single amnesic, multiple amnesic, single non-amnesic and multiple non-amnesic domains impaired, as described in a previous study [28].

Symptoms of depression were determined according to the Korean version of the 30-item Geriatric Depression Scale (GDS-K) [40] and the Korean version of the PHQ-9 [41]. The geriatric quality of life scale (GQOL) [42] and the Mini International Neuropsychiatric Interview (MINI) [43] were also administered to the participants. The final diagnosis was based upon a consensus meeting, attended by at least a neurologist, neuropsychologist, psychiatrist, and specialized nurse, where all the available clinical data and the results of the ancillary investigations were reviewed.

### Physical examination and interview

Each study participant received a comprehensive physical examination and completed an interviewer-administered questionnaire. Detailed data on sociodemographic factors and personal characteristics were collected including age, income, occupation, marital status, and education. Information on individual and familial medical history including dementia, depression, hypertension, diabetes, dyslipidemia was collected, as was information relating to other lifestyle factors including smoking status, alcohol intake, and physical activity [14,18]. To ensure uniformity, all interviewers underwent two days of training before the survey was initiated and sociodemographic data collected. McNemar's test was used to assess inter-surveyor reliability; no significant differences were seen (P > 0.05) [15].

### Blood pressure

Blood pressure measurements were performed with a mercury sphygmomanometer in a standardized cuff size adjusted to the circumference of the arm. The arm was placed with the cuff at the level of heart. Blood pressure was recorded using the means of 2nd and 3rd measurements taken after 5 minutes of rest in the supine position. Systolic and diastolic blood pressures were defined according to Korotkoff sounds I and V.

### Anthropometric measurements

The anthropometric measurements of each participant were taken after an overnight fast and while wearing light clothing and no shoes. Height was determined using a fixed wall-scale measuring device and was measured to the nearest 0.1 cm. Weight was determined within 0.1 kg for each subject using an electronic scale that was calibrated prior to each measurement. Body mass index (BMI) was calculated as weight in kilograms divided by the square of height in meters.

The waist-hip ratio (WHR) and waist circumference (WC) were used to assess android and central obesity. WC was measured over the umbilicus between the lower border of the ribs and the iliac crest in a horizontal plane. Hip circumference was measured over the widest point of the buttocks. Both the waist and hip circumference were measured twice and recorded to the nearest 0.5 cm. If the variation between these two measurements was greater than 2 cm, then a third measurement was taken and the mean was calculated by using the two closest recorded measurements. WHR was obtained by dividing the WC by the hip circumference.

The percent body fat (PBF) indicates the percentage of body fat to body weight. Bioelectrical impedance analysis was used to measure the PBF [44]. The PBF measurement that is derived from a bioelectrical impedance analysis is less accurate than measurements obtained by dual-energy X-ray absorptiometry. However, this method of estimating body composition has become increasingly popular because it is easy to use, non-invasive, relatively inexpensive, provides an accurate method for the assessment of body composition, and can be performed across a wide range of subjects with regard to age and body shape [45]. PBF was obtained by using segmental bioelectrical impedance with eight tactile electrodes according to the manufacturer's instructions (InBody 520; Biospace, Seoul, Korea). Participants stood upright on the foot electrodes with their arms held vertically while loosely gripping the hand electrodes. In this manner, the eight tactile electrodes were placed in contact with the thumb and palm of each hand and the front and rear soles of each foot. The microprocessor-controlled switches and the impedance analyzer were used to measure the segmental resistances of the arms, trunk, and legs without accounting for fluid redistribution. Alternating currents with a magnitude of 100 μA and frequencies of 5 to 500 kHz were used.

The bone mineral density (BMD; g/cm^2^) of the lumbar spine (L2-L4) was measured by dual-energy X-ray absorptiometry (DEXA) using DPX-MD+ (General Electric, Madison, Wis, United States). All scans performed on the same machine by the same operator were analyzed with the same software. The results were reported as a T score, a standard deviation measure related to peak bone mass for a young adult.

### Laboratory procedures

Baseline blood samples were taken after an overnight fast. Blood samples were collected and delivered to the Seoul Clinical Laboratories (Seoul, Korea) for blood assays. Laboratory tests included complete blood count, thyroid function test, liver function test, BUN/Cr, VDRL, vitamin B12, folate, blood sugar, lipid profile, homocysteine, C-reactive protein, and genotyping of the APOE polymorphism. These laboratory analyses were determined using commercially available kits. We classified the APOE ε4 polymorphism as present (homozygous or heterozygous) or absent. All measurements were obtained in a blind manner to each other and to the diagnosis.

### Carotid intima-media thickness (IMT)

Extracranial IMT measurements were performed according to a predetermined, standardized scanning protocol using high-resolution B-mode ultrasonography with a 7.5-MHz linear-type probe (GM-2600A, Panavista, Matsushita Co., Japan), as described previously [46]. Briefly, an experienced technologist who was blinded to the participant's clinical data made all ultrasound measurements. The IMT was evaluated as the distance between the luminal-intimal interface and the medial-adventitial interface, and it was measured with the use of an electronic caliper on the frozen frame of a suitable longitudinal image. Six to eight measurements of far-wall IMT were taken manually using ultrasound calipers, and the average values of these readings were used in the analyses. The mean and maximum of left and right IMTs were used in the analyses.

### Arterial Stiffness

Arterial stiffness was assessed by brachial-ankle PWV (baPWV) on both sides using an automated system (VP2000, Colin Medical Technology, Japan), using previously published methods [47]. In brief, blood pressure was checked in a sitting position after 10 min of rest before measurement of PWV. Electrocardiograms, bilateral brachial and ankle blood pressures, and carotid and femoral arterial pulse waves were measured simultaneously. Ten-second-interval bilateral brachial and posterior tibial arterial pressure waveforms were recorded using extremity cuffs connected to a plethysmographic sensor and an oscillometric pressure sensor wrapped around both arms and ankles. PWV was calculated from the distance between the two arterial recording sites divided by transit time. The time delay between the right brachial and posterior tibial arteries was obtained. The path lengths from brachial arteries to the ankle and the baPWV were calculated automatically. The validity and reliability of the automated device for measuring baPWV have been established previously [47].

Ankle-brachial index (ABI) was used as a measure of occlusive disease to the lower extremities. Pressures were measured in the right arm and both ankles (posterior tibial artery) automatically. Systolic blood pressure (SBP) in the ankle was divided by the SBP in the arm to create an ABI separately on each side.

### Definitions

Hypertension was defined by pre-admission history, medical records, and the criteria established by the Seventh Joint National Committee on Detection, Evaluation, and Treatment [48].

Diabetes was defined by the American Diabetes Association (ADA) criteria using blood glucose measurements [49]. Subjects with fasting blood glucose ≥ 126 mg/dL (6.99 mmol/L) and or 2-h blood glucose of ≥ 200 mg/dL (11.10 mmol/L) were classified as having diabetes. Subjects were also considered to be diabetic if they reported a history of diabetes diagnosed by a physician or if they were on anti-hyperglycemic agents irrespective of their blood glucose values.

Dyslipidemia was judged as present when laboratory examination of the serum at presentation showed a high total cholesterol level of >220 mg/dL (5.69 mmol/L), a high triglyceride level of >150 mg/dL (1.71 mmol/L), a low high-density-lipoprotein cholesterol level of <40 mg/dL (1.03 mmol/L), or when a history of treatment was present.

An ABI of ≤ 0.90 on at least one side was considered to be evidence of occlusive disease such as peripheral arterial disease.

### Role of the funding source

The funding sources had no role in data collection, data analysis, data interpretation, or the writing of this article. The corresponding author had full access to all the data in the study and had final responsibility for the decision to submit the article for publication.

## Results

The distribution of the study population is presented in Table 1. The distribution of age and gender was not different from that of the target population. In the screening study, the mean age was 68.38 ± 6.06 years and 56.1% were women. In the first-wave study, the mean age was 69.28 ± 5.44 years (67.33 ± 5.49 years when the screening study was performed) and 57.4% were women. In the second-wave study, the mean age was 70.95 ± 5.11 years (66.82 ± 5.17 years when the screening study was performed) and 54.8% were women. The distributional structure of age and gender from the screening test to the second-wave study did not differ significantly between studies (P > 0.05).

**Table 1:** Demographic characteristics of subjects

	Target population in Ansan-city			Baseline subjects		
		
	Total	Men	Women	Total	Men	Women
	36735	14677	22058	2767	1213	1554
		
Age (years)						
60–64	12814 (34.9)	5771 (39.2)	7043 (31.9)	912 (33.0)	425 (35.0)	487 (31.3)
65–69	10386 (28.3)	4175 (28.5)	6211 (28.2)	774 (28.0)	327 (27.0)	447 (28.8)
70–74	6837 (18.6)	2494 (17.0)	4342 (19.7)	553 (20.0)	249 (20.5)	304 (19.6)
75–79	4353 (11.8)	1436 (9.8)	2917 (13.2)	348 (12.6)	144 (11.9)	204 (13.1)
80–84	2346 (6.4)	801 (5.5)	1545 (7.0)	180 (6.5)	68 (5.6)	112 (7.2)

## Discussion

The AGE study was designed to obtain comprehensive epidemiological data on the health status and common geriatric diseases of elderly Koreans and to establish a comprehensive information database on public health and functional status in elderly Koreans. Strengths of the study are that the participants were randomly selected from a defined population in Korea. This study was well designed with stratified randomized sampling methods, representing a geographically-defined Korean population. All participants were assessed using structured instruments for geriatric disease and precise neuropsychological tests. However, this study also has some limitations. No institutionalized elderly participants were included in this study therefore this sample population might not be completely representative of the geriatric Korean population. Furthermore, the study's cohort was not large enough to estimate the prevalence of low-level geriatric diseases. Additional recruitment will be performed at each follow-up stage to increase sub-populations in areas of interest or to refresh the AGE cohort as it ages.

Longitudinal follow-up is ongoing and the comprehensive health data obtained is expected to contribute to policy formulation and planning of health, welfare management, and other social services for elderly persons in Korea.

## Competing interests

The authors declare that they have no competing interests.

## Authors' contributions

All authors contributed to the development and writing of the protocol. All authors have been involved in the drafting and revision of this manuscript and have given approval of the final manuscript.

## Pre-publication history

The pre-publication history for this paper can be accessed here:


